# Non-invasive Method for Collection of *Clarias magur* Spermatozoa for Breeding

**DOI:** 10.1016/j.mex.2020.100929

**Published:** 2020-05-23

**Authors:** Sullip Kumar Majhi, Pradip Kumar Maurya, Santosh Kumar, Vindhya Mohindra, Kuldeep Kumar Lal

**Affiliations:** ICAR-National Bureau of Fish Genetic Resources, Canal Ring Road, Dilkhusa P.O., Lucknow 226002, Uttar Pradesh, India

**Keywords:** Catfish, Surgery, Spermatozoa, Breeding, Aquaculture

## Abstract

Induced spawning is more inefficient in *Clarias magur* than in other fish species such as cyprinids and salmonids. Ovulation can be induced in the female *C. magur* by using pituitary extract or synthetic hormones. However, milt from the male *C. magur* cannot be obtained by hand stripping because the volume of seminal fluid in the testes is extremely low. Notably, similar problems are observed in other male catfishes such as *C. gariepinus* and *C. lazera*. Because milt from the males cannot be obtained for use in artificial fertilization of eggs, males are invariably sacrificed, and the testicular tissue is excised and macerated to obtain spermatozoa.

• We developed an alternative approach that allowed harvesting of *C. magur* spermatozoa through surgery for artificial fertilization without sacrificing male fish.
• The surgically obtained spermatozoa were used to inseminate *C. magur* eggs; the cross resulted in healthy progeny with a fertilization rate of 80%–98% and hatching of up to 85% of fertilized embryos; similar to those obtained using the conventional sacrificial approach (hatching percentage range of 45%–85%) [1].
• This indicated the viability of partial surgical harvest of testicular tissue in seed production in *C. magur* for aquaculture without sacrificing male fish.

## Graphical Abstract

**A**) Reproductive mature *Clarias magur* (scale bar = 6 cm). **B**) Surgery of *C. magur* under anaesthetic condition (100 ppm 2-phenoxyethanol) for partial harvest of testicular tissue. **C**) Collection of spermatozoa from the harvested testicular tissue. **D**) Cryopreservation of harvested spermatozoa for posterity. **E**) Artificial insemination of *C. magur* eggs by freshly harvested spermatozoa. **F**) Recovery of cryopreserved spermatozoa, quantification, viability assessment and artificial insemination with freshly derived eggs from *C. magur* female. **G**) Generation of *C. magur* progeny.

**Figure:**
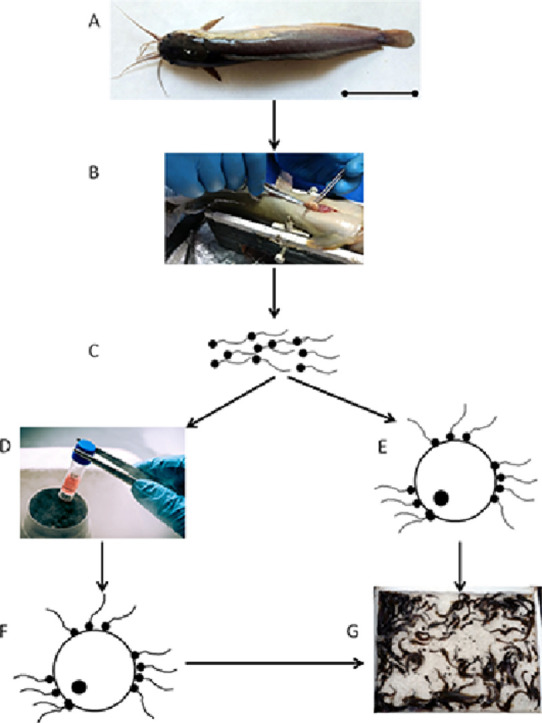

Specifications Table

**Table:** 

Subject Area:	Agricultural and Biological Sciences
More specific subject area:	*Reproductive Biotechnology*
Method name:	*Non-invasive method for collection of Clarias magur spermatozoa*
Name and reference of original method:	*This is the first report on collection of catfish Clarias magur spermatozoa by non-invasive method.*
Resource availability:	*Not applicable*

## Method details

**Fig. 1:**
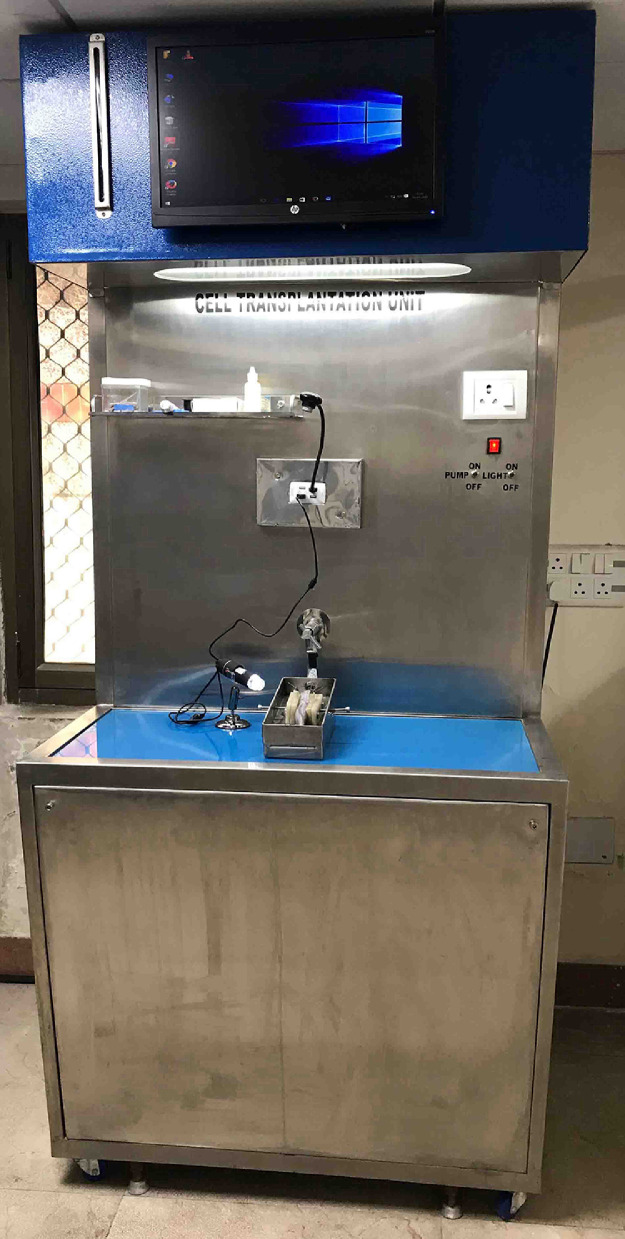
View of surgery table. The surgery table has an inlet and outlet for water flow. Water containing the anaesthetic agent from the lower tank was raised to the upper tank by using a water pump; the water descended under gravity, passed through the operculum of the fish that was placed inside the chamber.

**Fig. 2:**
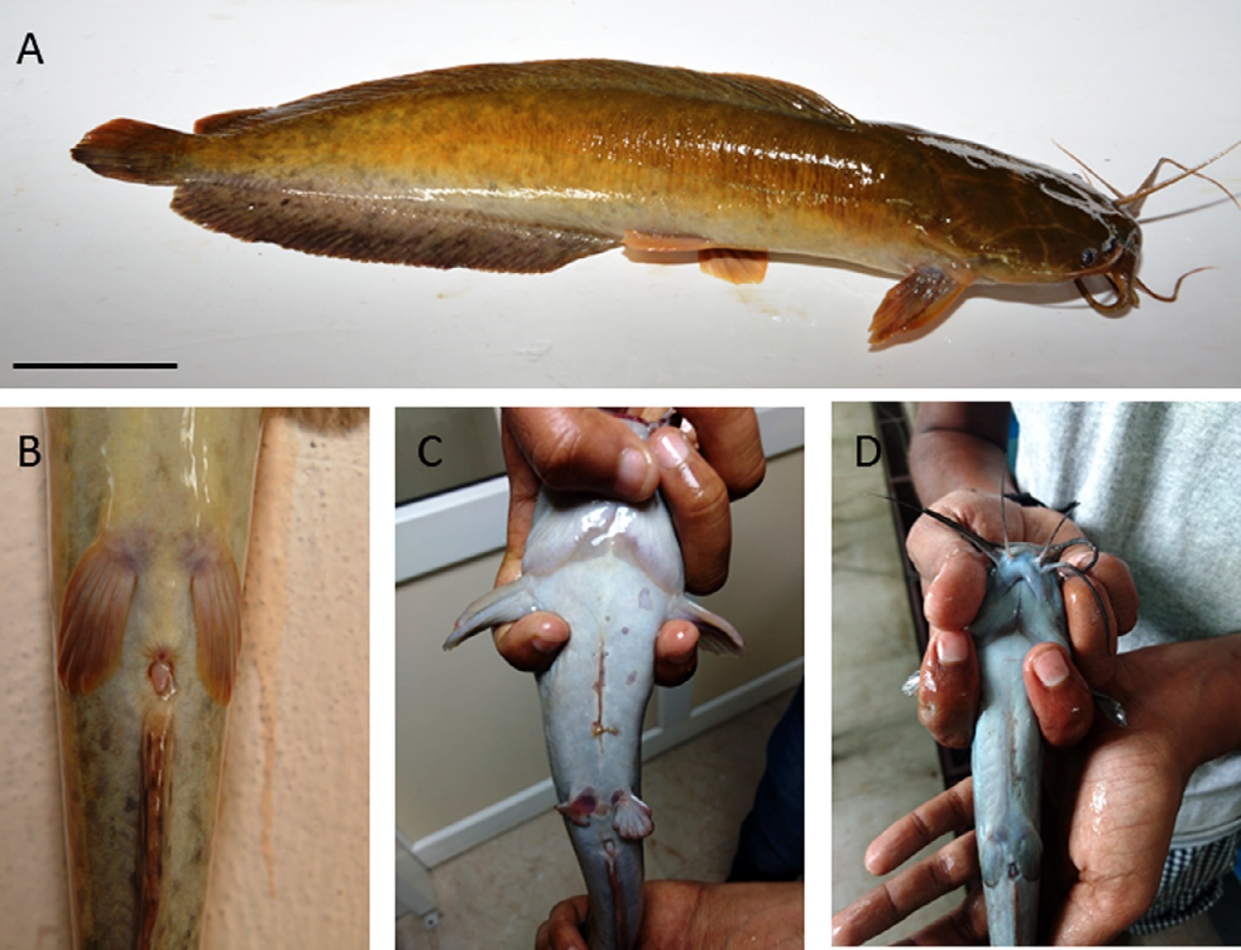
Healing of surgery wound. A, B) View of Clarias magur before surgery. C) Partial healing of surgery wound two weeks after the procedure. D) Complete healing of surgery wound four weeks after the procedure. Scale bar indicates 2.5 cm (A).

Feeding to the catfish *Clarias magur* (n=15; size: 210 ± 10.5 g; age: 18 months) were stopped 48 hr before the surgery. The fish were first anaesthetized using 200 ppm of 2-phenoxy ethanol, and then place onto the surgery table (Fig. 1). During the surgery, attention was given to maintain the water quality parameter (temperature: 25°C; dissolved oxygen: 5.5 ppm; pH: 7.5; hardness: 40 ppm), mainly to reduce stress to the animal. Adequate care was taken to prevent desiccation by covering the body with wet tissue paper, only exposing the abdominal region. Sterile scalpel blade was used to make a 2.5 cm long midline incision in the abdomen [2]. The cut abdominal muscle was gently stretched from both sides by using forceps. The testicular lobes were carefully lifted from coelomic cavity with a soft spatula. A small portion of testicular tissue from both the lobe (up to 25% of total testicular tissue from each lobe) was carefully excised by using sterile scissors and forceps, without damaging the efferent duct and any other adjacent internal organs. The excised tissue was immediately stored in physiological saline solution (0.85% NaCl). Then the abdominal incision was stitched using a sterile surgical needle and thread (Needle: 3/8 circle, 15 mm; thread: catgut suture USP size 5/0, 2-matric). The surgical wound was topically treated with an antimycotic solution to avoid secondary infection. Then the fish was resuscitated in clean water (video). On completion of the surgical procedure, the fish was kept in a tank containing 0.2% saline water and 10 ppm Potassium permanganate and provide vigorous aeration for 15 minutes. In this condition fish recover from the anesthesia within 1-2 minutes. Then animals were transferred to another tank containing only freshwater with mild aeration. The feeding to the animal was resumed after 24–-36 hr until they began to swim normally, similar to as control animals (non-surgery). On resumption of feeding, the fish were transferred to a bigger tank and observe for healing of the surgery wound (Fig. 2). On complete healing, fish were stocked in a 1000- L capacity FRP tank with a 10- cm soil bed for testicular regeneration. During this phase, besides commercial diet, the fish were fed with zooplanktons Rotifera, Cladocera and Copepoda every day.

## Declaration of Competing Interest

The authors declare that they have no known competing financial interests or personal relationships that could have appeared to influence the work reported in this paper.

## Appendix Supplementary materials

Supplementary material *and/or* Additional information: *None* Image, video 1
